# Childhood exposure to low/moderate dose ionizing radiation and risk of early‐onset dementia: A Tinea Capitis Cohort study

**DOI:** 10.1002/alz70860_106532

**Published:** 2025-12-23

**Authors:** Angela Chetrit, Nomi Carlebach, Galit Hirsh‐Yechezkel, Ilya Novikov, Michal Schnaider Beeri, Samuel M. Goldman, Kristine Yaffe, Raquel C. Gardner

**Affiliations:** ^1^ Gertner Institute for Epidemiology and Health Policy, Ramat Gan, Israel, Israel; ^2^ Herbert and Jacqueline Krieger Klein Alzheimer's Research Center, Piscataway, NJ, USA; ^3^ University of California San Francisco / San Francisco Veterans Affairs Medical Center, San Francisco, CA, USA; ^4^ Global Brain Health Institute, Memory and Aging Center, University of California San Francisco, San Francisco, CA, USA; ^5^ University of California San Francisco / San Francisco VA Medical Center, San Francisco, CA, USA; ^6^ Sheba Medical Center, Joseph Sagol Neuroscience Center, Ramat Gan, Israel

## Abstract

**Background:**

It is unclear whether childhood exposure to low‐to‐moderate dose ionizing radiation (LMIR) is associated with later‐life neurocognitive deficits or dementia. We studied the association of childhood LMIR with early‐onset dementia (EOD), defined as all‐cause dementia diagnosed until age 65 years, in the unique Tinea Capitis Cohort (TCC) in Israel.

**Method:**

The TCC comprised children (ages 0‐15 years) who received head and neck irradiation for treatment of a benign scalp infection from 1946‐1960 and age‐matched non‐irradiated sibling and population controls. Data on outcomes was obtained from the largest national health fund that insures approximately 70% of the TCC from 1998‐2016 including the main outcome of EOD and covariates of hypertension, coronary artery disease, diabetes, cerebrovascular disease, epilepsy and depression. We used Cox proportional hazard models to estimate the association between LMIR exposure and EOD with age as time‐scale. Models were adjusted for competing risk of death using the Fine‐Gray method and additionally adjusted for sex, socioeconomic status, and the listed health covariates. Models were estimated for the entire cohort and by age‐at‐irradiation strata (<5, 5‐9, 10‐15 years).

**Result:**

The linked cohort included *N* = 17,824 beneficiaries (7,395 irradiated; 10,429 non‐irradiated). Among the irradiated, 23%, 53% and 24% were irradiated at age 0‐4 years, 5‐9 years, and 10‐15 years, respectively. The mean (standard deviation) and range of estimated brain irradiation dose was 151 (51), 98‐557 cGy. Average age at end of follow‐up was 63.9 years for irradiated; 63.7 years, non‐irradiated. There were 107 participants with EOD (0.62% of irradiated; 0.58%, non‐irradiated) and 1,190 deaths (7.29% of irradiated; 6.24%, non‐irradiated). In the total cohort, there was no association between LMIR and EOD (hazard ratio [HR], 95% confidence interval [95%CI]: unadjusted 1.04, 0.71‐1.52; fully‐adjusted 0.91, 0.62‐1.33). However, among those irradiated at age 10‐15 years, there was a significant association between LMIR and increased risk of EOD (HR, 95%CI: unadjusted 2.82, 1.46‐5.42; fully‐adjusted 2.40, 1.27‐4.55).

**Conclusion:**

In this cohort, children irradiated at age 10‐15 years had elevated risk of EOD compared to non‐irradiated controls. This finding highlights the potential long‐term neurodegenerative sequelae of childhood LMIR and deserves further research into mechanisms and prevention.

